# Nutrition education for healthcare professionals in Ireland: insights from curriculum, accreditation and registration standards

**DOI:** 10.1017/S1368980025101705

**Published:** 2026-01-09

**Authors:** Gemma McMonagle, Lisa Ryan, Rónán Doherty, Laura Keaver

**Affiliations:** 1 Department of Health and Nutritional Sciences, Atlantic Technological University, Ash Lane, Sligo, Co. Sligo F91 YW50, Ireland; 2 Department of Sport, Exercise and Nutrition, Atlantic Technological University, Dublin Road, Galway H91 T8NW, Ireland; 3 Department of Tourism and Sport, Atlantic Technological University, Port Road, Letterkenny, Co. Donegal F92 FC93, Ireland

**Keywords:** Nutritional management, Education, Healthcare professionals, Curriculum

## Abstract

**Objective:**

Nutrition plays a valuable role in health promotion and disease prevention. Nutrition education for healthcare professionals (HCPs) has been widely explored globally. However, it has not been investigated extensively within Ireland. This research aimed to assess references to nutrition within education programmes, accreditation curricula standards and registration requirements of professional bodies for primary care and community HCPs in Ireland.

**Design:**

A cross-sectional content analysis was conducted. Data collection was carried out in October 2024.

**Setting:**

Ireland

**Participants:**

A sample of primary care and community HCPs was included (*n* 10). An online search identified education programmes (undergraduate and postgraduate), accreditation curriculum standards and registration requirements from professional bodies governing primary care and community HCPs. Relevant webpages and documentation were reviewed to determine direct references to nutrition (e.g. ‘diet’, ‘nutrition’, ‘eating’, ‘food’) and/or indirect references to nutrition (e.g. ‘health promotion’ and ‘well-being’).

**Results:**

Out of fifty-two education programmes, 26·9 % (*n* 14) made direct reference to nutrition, with the majority (*n* 8) of these being postgraduate level. Furthermore, 20 % (*n* 2) of the HCP bodies referred directly to nutrition within their registration requirements (one of which was for dietitians), and 50 % (*n* 5) referred directly to nutrition within their accreditation standards.

**Conclusions:**

This research demonstrates a sparsity of nutrition within key education standards for primary care and community HCPs in Ireland. Key recommendations include a call to action for formal and consistent embedding of nutrition within education for medical professionals in Ireland, in line with international best practice.

Malnutrition directly impacts clinical outcomes; therefore, medical doctors and healthcare professionals (HCPs) have been urged to recognise the importance of nutrition care^(1)^. However, the inadequacy of nutrition education for medical students is a barrier to the provision of nutrition care globally^(2)^. Medical students were not confident in nutrition competencies^(3)^, which led to calls for additional nutrition education for medical students in the UK^(4,5)^. A review of international medical education (i.e. for medical doctors) found that less than half of accreditation requirements and curriculum guidance documents included reference to nutrition^(6)^. This prompted further calls to action for a top-down approach to regulate the inclusion of nutrition within medical education^(7)^. Notably, previous nutrition education has been linked to greater knowledge, skills, communication and attitudes towards nutrition care amongst a group of pharmacists in Ireland^(8)^.

In many countries, steps have been taken to improve the provision of nutrition education for HCPs. Australia has prioritised embedding prevention in primary health care and recognised nutrition as a key component of health^(9)^. The Association for Nutrition (AfN), the accreditation body for nutrition programmes, has developed an undergraduate nutrition curriculum for medical doctors in the UK^(10)^. An interprofessional working group devised the AfN curriculum to facilitate the integration of nutrition into undergraduate medical education^(11)^. The Medical Council of Ireland’s accreditation process complies with the World Federation for Medical Education’s Global Standards for Quality Improvement^(12)^. Irish medical education providers use the standards as a framework for their curriculum development, but there is no stipulation for the inclusion of nutrition.

Overall, the evidence base illustrates a lack of nutrition in medical education internationally^(2)^, yet there is a paucity of research assessing the situation in Ireland. Qualitative research cites a lack of knowledge^(13)^ and inadequate training in nutrition^(14)^ as barriers to the provision of nutrition care by HCPs in Ireland. Confidence in nutrition knowledge and skills was low amongst a group of 206 HCPs surveyed in Ireland, yet the majority expressed a need for further nutrition education in their role^(15)^. Preliminary findings suggest that reference to nutrition is sparse in HCPs’ education in Ireland^(16)^. Therefore, this research aimed to assess the level of nutrition within education for primary care and community HCPs in Ireland – education programmes, accreditation curricula standards and requirements for registration with professional bodies were assessed for reference to nutrition. The present research specifically focuses on primary care and community HCPs in Ireland, given the high frequency of contact with the general population^(17)^. In addition, in the absence of a national curriculum for medical education, the curriculum for GPs in Ireland was compared against the AfN UK undergraduate curriculum in nutrition for medical doctors^(10)^. This comparative analysis was conducted to allow provisional insight into the applicability of the curriculum in the Irish setting.

## Methods

This study adhered to the Strengthening the Reporting of Observational Studies in Epidemiology (STROBE) statement^(18)^.

### Study design

This cross-sectional content analysis was conducted in three phases. Phase 1 considered the level of nutrition within undergraduate (primary degree) and postgraduate (after completion of primary degree) education programmes for primary care and community HCPs in Ireland. Ten primary care and community HCPs were considered for the purposes of this research; this included the general practitioner (GP), nurse, public health nurse, occupational therapist, podiatrist, psychologist, speech and language therapist, dietitian, physiotherapist and dentist. Phase 2 reviewed (a) the accreditation curriculum standards for education programmes and (b) the registration requirements for professional bodies of the ten HCPs for references to nutrition within their documentation, that is, direct references to nutrition (e.g. ‘diet’, ‘nutrition’, ‘eating’, ‘food’) and indirect references to nutrition (e.g. ‘health promotion’, ‘well-being’)^(5)^. Phase 3 compared the nutrition curriculum statements outlined in the AfN curriculum for medical doctors^(10)^ with the curriculum for GPs in Ireland^(19)^.

### Data collection

Data for phases 1–3 were collected from online searches in October 2024 and verified by a second researcher. A list of HCPs, relevant national education programmes and their professional bodies was retrieved from the national health service webpage^(20)^. In phase 1, the webpages of the identified HCP education programmes (undergraduate and postgraduate) were consulted. All education programmes available at the time of data collection were included. The webpages were scanned for any information referring to nutrition, both direct (e.g. nutrition, diet, food) and indirect (e.g. breastfeeding, well-being, health promotion), the level of inclusion of nutrition (within programme description/module component/placement component) and whether the nutrition component was mandatory/optional.

In phase 2(a), the webpages for the professional bodies that regulate the accreditation curriculum standards for primary care and community HCP education programmes were consulted. The curricula were retrieved from the webpages of the professional bodies. The document was scanned by the researcher, and the search function was used to determine direct references to nutrition (e.g. nutrition) and indirect references to nutrition (e.g. well-being). Similarly, in phase 2(b), the webpages of professional bodies governing the registration requirements of primary care and community HCPs were consulted. Any documentation relating to requirements or standards of proficiency for professional registration was scanned by the researcher, and the search function was used to determine direct references to nutrition (e.g. nutrient) and indirect references to nutrition (e.g. breastfeeding).

In phase 3, the AfN’s Curriculum for Medical Education^(10)^ was used as a benchmark for eight key topic areas to be addressed in medical education. The curriculum for GPs in Ireland^(19)^ was compared to the AfN curriculum topic areas^(10)^ to identify any alignments in nutrition-related terminology. The document was reviewed by the researcher to identify references to any of the competency statements, and the search function was then used to search for key terms within the competency statements such as ‘nutrition’, ‘hydration’ and ‘malnutrition’.

### Data analysis

The data were recorded on a spreadsheet and collated into tables to illustrate the findings of each phase. The data were stratified by HCP. The outcomes of this research included the frequency of reference to nutrition, both directly and indirectly, and comparisons between the curriculum documents from AfN^(10)^ and ICGP^(19)^.

## Results

### Reference to nutrition within education programmes

A total of fifty-two education programmes, undergraduate (*n* 22) and postgraduate (*n* 30), for primary care and community HCPs were considered for this research. For some specialist professions, only postgraduate training was available (i.e. GPs, public health nurses and psychologists). All education programmes (100 %) for medical doctors, GPs, practice nurses, podiatrists, speech and language therapists and dietitians contained a nutrition component (i.e. either direct or indirect reference to nutrition). However, only 26·9 % (*n* 14) of programmes made direct reference to nutrition, with the majority (*n* 8) of these being postgraduate programmes (e.g. ‘Demonstrate ability to explain to patients the long-term impact on health of… poor diet…’ (GP programme)).

A further 38·5 % (*n* 20) of programmes referred to nutrition indirectly (e.g. by referring to ‘health promotion’ or ‘well-being’). The majority of nutrition references were at module level, that is, either within a module title, description or learning outcome (65·4 %; *n* 34). Nutrition references were generally within mandatory components of programmes; however, some programmes for practice nurses, public health nurses and physiotherapists contained nutrition at an optional level (Table 1).

**Table 1.** Reference to nutrition within third-level education programmes for primary care and community healthcare professionals in Ireland

Healthcare professionals	Education level	Programmes available, *n*	Programmes with nutrition component		Programmes which reference nutrition, *n*		Level of inclusion, *n*			Mandatory or optional	Example of direct reference to nutrition	Example of indirect reference to nutrition
			*n*	%	Directly	Indirectly	Programme description	Module component	Placement			
General practitioner (general medicine – prerequisite)	Undergraduate	6	6	100	4	2	2	6	1	Mandatory	Obesity	Disease aetiology
General practitioner specialist training	Postgraduate	1	1	100 %	1	0		1		Mandatory	Demonstrate ability to explain to patients the long-term impact on health of… poor diet.	
Practice nurse	Undergraduate	3	3	100	0	3		3		Mandatory		Health promotion
	Postgraduate	2	2	100	0	2		2		Both		Diabetes
Public health nurse	Undergraduate	0	0		0	0						
	Postgraduate	3	2	67	0	3		3	1	Both		Well-being
Occupational therapist	Undergraduate	3	1	33	1			1		Mandatory	Healthy diet	
	Postgraduate	0	0		0	0						
Podiatrist	Undergraduate	1	1	100	0	1	1	1		Mandatory		Diabetes
	Postgraduate	0	0		0	0						
Psychologist	Undergraduate	0	0		0	0						
	Postgraduate	14	3	21	0	3		3		Mandatory		Health promotion
Speech and language therapist	Undergraduate	3	3	100	0	3		3	3	Mandatory		Swallowing science
	Postgraduate	1	1	100	1			1	1	Mandatory	Feeding	
Dietitian	Undergraduate	1	1	100	1	0	1	1	1	Mandatory	Nutrition science	
	Postgraduate	3	3	100	3	0	3	3	3	Mandatory	Dietetic assessment	
Physiotherapist	Undergraduate	3	1	33	0	1		1		Both		Personal training
	Postgraduate	5	4	80	3	1	2	4		Both	Nutrition	
Dentist	Undergraduate	2	1	50	0	1		1		Mandatory		Aetiological factors
	Postgraduate	1	0		0	0						

### Reference to nutrition within registration requirements

Table 2a lists the professional bodies and their respective documents that convey the registration requirements for the ten HCPs investigated within this study. Of the ten HCPs, only 20 % (*n* 2) referred directly to nutrition within their registration requirements (i.e. Dietitian and Speech and Language Therapist). A further 50 % of HCPs (*n* 5) had indirect reference to nutrition within their registration requirements (e.g. dentist referred to ‘oral health’). There was no reference to nutrition within registration requirements for the GP, practice nurse or public health nurse.

**Table 2a.** Reference to nutrition within the registration requirements for primary care and community healthcare professionals in Ireland

Healthcare professional	Professional body	Document details	Document URL	Reference to nutrition (direct/indirect/no mention)	Example
General practitioner	Irish Medical Council	Irish Medical Council Registration Applications webpage – Requirement to be registered with the Irish College of General Practitioners or equivalent evidence of qualifications.	https://www.medicalcouncil.ie/registration-applications/first-time-applicants/	None	
Nurse	Nursing and Midwifery Board of Ireland (NMBI)	Scope of Nursing and Midwifery Practice Framework	https://www.nmbi.ie/nmbi/media/NMBI/Publications/Scope-of-Nursing-Midwifery-Practice-Framework.pdf?ext=.pdf	None	
Public health nurse	Nursing and Midwifery Board of Ireland (NMBI)	Scope of Nursing and Midwifery Practice Framework	https://www.nmbi.ie/nmbi/media/NMBI/Publications/Scope-of-Nursing-Midwifery-Practice-Framework.pdf?ext=.pdf	None	
Occupational therapist	CORU – Occupational Therapists Registration Board	Occupational Therapists Registration Board Standards of Proficiency for Occupational Therapists	https://coru.ie/files-education/otrb-standards-of-proficiency-for-occupational-therapists.pdf	Indirect	‘Demonstrate a critical understanding of relevant biological sciences including …a knowledge of health and well-being…’
Podiatrist	CORU – Podiatrists Registration Board	Podiatrists Registration Board Standards of Proficiency for Podiatrists.	https://coru.ie/files-education/podiatrists-registration-board-standards-of-proficiency.pdf	Indirect	‘foot health promotion’ ‘health and well-being’; ‘biological sciences’
Psychologist	Psychological Society of Ireland	The Psychological Society of Ireland – Standards on the Accreditation of Undergraduate and Conversion Programmes	https://www.psychologicalsociety.ie/source/Accreditation/Undergraduate%20Accreditation%20Standards%202019update_file_1161%20(3).pdf	Indirect	‘Biological psychology’, ‘Physical and mental health’
Speech and language therapist	CORU – Speech and Language Therapists Registration Board	Standards of Proficiency and Practice Placement Criteria	https://coru.ie/files-recognition/standards-of-proficiency-for-speech-and-language-therapists.pdf	Direct	‘Demonstrate an understanding of… the potential impact of an impairment in eating/drinking on health, quality of life and well-being’
Dietitian	CORU – Dietitians Registration Board	Standards of Proficiency for Dietitians	https://coru.ie/files-education/drb-standards-of-proficiency-for-dietitians.pdf	Direct	‘Know and understand the core elements of nutrition and dietetic practice’
Physiotherapist	CORU – Physiotherapists Registration Board	Standards of Proficiency for Physiotherapists	https://coru.ie/files-recognition/standards-of-proficiency-for-physiotherapists.pdf	Indirect	‘knowledge of health, disease, disorder dysfunction and health promotion’
Dentist	Dental Council of Ireland	Code of Practice: Professional Behaviour and Ethical Conduct Dental Council of Ireland	https://www.dentalcouncil.ie/wp-content/uploads/2023/06/Code-of-Practice-Professional-Behaviour-and-Ethical-Conduct-20220301.pdf	Indirect	‘health’, ‘oral health’

### Reference to nutrition within accreditation curriculum standards

Table 2b conveys details of the professional bodies that set out the accreditation curriculum standards for HCPs. Half (*n* 5) of the curricula standards made direct reference to nutrition, a further 40 % (*n* 4) made indirect reference to nutrition and one (10 %) document made no reference to nutrition (Psychologists).

**Table 2b.** Reference to nutrition within the accreditation curriculum standards for primary care and community healthcare professionals in Ireland

Professional body	Details	Document URL	Reference to nutrition (direct/indirect/no mention)	Example
The Irish College of General Practitioners	ICGP Curriculum for general practitioner training in Ireland for 2020	https://www.irishcollegeofgps.ie/Home/Training-Assessment/Trainee-Hub/Curriculum	Direct	‘Demonstrate a knowledge of the impact of long-term illness on nutrition’.
Nursing and Midwifery Board Of Ireland (NMBI)	Graduate Entry General Nurse Registration Education Programme Standards and Requirements.	https://www.nmbi.ie/NMBI/media/NMBI/NMBI-Graduate-Entry-Nursing-Standards-Requirements-First-Edition_1.pdf?ext=.pdf	Direct	‘Principles of nutrition and dietetics’.
Nursing and Midwifery Board Of Ireland (NMBI)	These standards and requirements set out the educational standards and requirements for public health nursing post-registration education programmes.	https://www.nmbi.ie/NMBI/media/NMBI/Public-Health-Nursing-Education-Programme-Standards-and-Requirements.pdf?ext=.pdf	Indirect	‘Health promotion’; ‘well-being’
CORU – Occupational Therapists Registration Board	Occupational Therapists Registration Board Criteria for Education and Training Programmes	https://coru.ie/files-education/otrb-criteria-for-education-and-training-programmes.pdf	Indirect	‘The curriculum must ensure that those who successfully complete the programme meet the standards of proficiency’.
CORU – Podiatrists Registration Board	Podiatrists Registration Board Criteria for Education and Training Programmes	https://coru.ie/files-education/podiatrists-registration-board-criteria-for-education-and-training-programmes.pdf	Indirect	‘The curriculum must ensure that those who successfully complete the programme meet the standards of proficiency’.
CORU – The Psychologists Registration Board	Education programmes are required to meet Standards of proficiency and Core Criteria – both in draft consultation.	Regulatory framework under development https://coru.ie/public-protection/public-consultations/current-consultations/psrb-2024-consultation-standards-e-book.pdf https://coru.ie/news/news-for-health-social-care-professionals/psrb-draft-core-criteria-for-education-and-training-programmes.pdf	None	None
CORU – Speech and Language Therapists Registration Board	Speech and Language Therapists Registration Board Criteria for Education and Training Programmes	https://coru.ie/files-education/sltrb-profession-specific-criteria-for-education-and-training-programmes.pdf	Direct	‘The curriculum must ensure that those who successfully complete the programme meet the standards of proficiency’.
Dietitians Registration Board at CORU	Dietitians Registration Board Criteria for Education and Training Programmes	https://coru.ie/files-education/drb-profession-specific-criteria-for-education-and-training-programmes.pdf	Direct	‘The curriculum must ensure that those who successfully complete the programme meet the standards of proficiency’.
CORU – Physiotherapists Registration Board	Code of Professional Conduct and Ethics for Physiotherapists devised by the Physiotherapists Registration Board at CORU	https://www.coru.ie/files-codes-of-conduct/prb-code-of-professional-conduct-and-ethics-for-physiotherapists.pdf	Indirect	‘Health’, ‘well-being’
Dental Council of Ireland	Dental Council Accreditation Manual	https://www.dentalcouncil.ie/wp-content/uploads/2024/08/Dental-Council-Accreditation-Manual-2022_to-Schools.pdf	Direct	‘Dietary and behavioural analysis’; ‘provide dietary advice’

### Comparison of Irish general practitioner curriculum standards with the Association for Nutrition curriculum standards for medical doctors

Out of eight curriculum statement topics, the GP curriculum standards addressed 37·5 % (*n* 3) and partly addressed a further 37·5 % (*n* 3) (see online supplementary material, Supplemental materials). Reference to ‘Nutrition screening and assessment’ or ‘Specific dietary requirements’ could not be found within these competency statements.

## Discussion

This study aimed to assess any reference to nutrition within education programmes, education curricula for accreditation standards and requirements for registration with professional bodies for primary care HCPs in Ireland. The curriculum for GPs in Ireland^(19)^ was compared against the AfN UK undergraduate curriculum in nutrition for medical doctors^(10)^. In summary, this research shows that just over a quarter of HCP education programmes made direct reference to nutrition, and the majority of these were postgraduate. Only two (20 %) of the HCP bodies referred directly to nutrition within their registration requirements, one of which was for dietitians. Half of the accreditation standards assessed referred directly to nutrition (*n* 5). Lastly, six out of eight topic areas from the AfN nutrition curriculum for medical doctors were either fully or partly addressed within the curriculum standards for GPs in Ireland; however, ‘nutrition screening and assessment’ or ‘specific dietary requirements’ were not included.

Overall, the findings of the present study concur with international data demonstrating a deficit of nutrition within medical education for HCPs. Prior to this study, a systematic review of the views of medical students and medical educators found that nutrition is insufficiently embedded in medical education internationally^(2)^. In the present study, many education programmes did not make direct reference to nutrition within their programme webpage, description, module or placement details. Unsurprisingly, nutrition education was positively associated with greater scores in knowledge, skills, communication and attitudes towards nutrition care amongst pharmacists in Ireland^(8)^. Previous research in Ireland amongst a group of GP trainees and programme directors agreed that it is within the role of the GP to promote a healthy diet but alluded to the fact that nutrition education was inadequate^(21)^, views similarly expressed in the UK^(4)^.

Nutrition has also been insufficiently represented in accreditation and curriculum guidance for medical education^(6)^. However, despite providing a comprehensive review of nutrition within medical education internationally, this research did not consider Ireland. The present study found that approximately half of accreditation curricula standards and a fifth of registration requirements for HCPs in Ireland made direct reference to nutrition. Previous research by Lepre *et al*. (2021) found that only 44 % of accreditation and curriculum guidance for medical doctors internationally included nutrition^(6)^. The current findings depict a clear deficit of nutrition components within the education regulatory framework for medical students in Ireland. The accumulation of evidence in relation to medical doctors and HCPs recognises the importance of nutrition care in clinical practice to address the global burden of malnutrition and non-communicable diseases^(7,8,15)^.

The introduction of the nutrition curriculum for undergraduate medical doctors in the UK has demonstrated a commitment to reform medical education^(11)^ and provided a benchmark for neighbouring countries. It is important to note that the curriculum for GPs in Ireland^(19)^ was developed for a postgraduate qualification for GP training, whereas the AfN curriculum is specifically developed for undergraduate medical doctors^(10)^. However, the absence of a national curriculum for undergraduate medical education in Ireland prevented a like-for-like comparison.

This study was the first in Ireland to investigate the level of nutrition within education programmes, accreditation standards and registration requirements. This research has provided a baseline comparison of the GP curriculum in Ireland with the nutrition curriculum for medical doctors in the UK. The main limitation of this research is the nature of data collection relying on online resources. The extent of this information varied considerably; therefore, future research should explore teaching activities and assessment details of the education programmes.

In conclusion, this research conveys that reference to nutrition within key education standards for primary care and community HCPs in Ireland is insufficient. The implications of these findings present a challenge, particularly for professional bodies involved in the governance of the HCPs and the regulation of the education providers. Future revisions of education programmes, accreditation curriculum standards and registration criteria for medical professionals within Ireland should include a thorough review of nutrition components. A fundamental recommendation from this research includes a call to action for formal and consistent embedding of nutrition within education for medical professionals in Ireland, in line with international best practice.

## Supporting information

McMonagle et al. supplementary material McMonagle et al. supplementary material

## Supplementary material

For supplementary material accompanying this paper, visit https://doi.org/10.1017/S1368980025101705.
