# Supplementary material for: Nutrition education for healthcare professionals in Ireland: insights from curriculum, accreditation and registration standards
Source: Public Health Nutr. 2026 Jan 9;29(1):e27. doi: 10.1017/S1368980025101705 (PMC12895439; doi:10.1017/S1368980025101705)
Supplement: McMonagle et al. supplementary material [file S1368980025101705sup001.docx]

**Supplementary table: Cross-comparison of Association for Nutrition's (UK) Curriculum Statement Topics with the Curriculum Standards for General Practitioners in Ireland**

| **Curriculum Statement Topics** | **Addressed in Curriculum Standards (Yes/ No/ Partly)** | **Examples of related learning outcome(s)/ competencies** |  |  |
| --- | --- | --- | --- | --- |
| Nutrition and hydration in health and disease | Partly | "Diagnose and manage… dehydration" | "Health promotion in relation to… nutrition…" | “Demonstrate a knowledge of the impact of long-term illness on nutrition” |
| Nutrition screening and assessment | No | mentions "health screening" | "Complete with confidence a dietary history relevant to a patient with or likely to develop CVD" |  |
| Effect of nutrition status on illness | Yes | "Initiate discussion with patients... diet and the link between these lifestyle issues and health" |  |  |
| Malnutrition: overweight, obesity and metabolic syndrome | Yes | "Nutritional problems (underweight, overweight, obesity, coeliac disease)" |  |  |
| Nutrition in health promotion and illness prevention (public health) | Partly | "Be aware of the factual elements underpinning health-promoting practice such as... health promotion in relation to... Nutrition..." |  |  |
| Hydration | Partly | "Diagnose and manage… dehydration" |  |  |
| Specific dietary requirements | No |  |  |  |
| Malnutrition: underweight/ undernourished | Yes | "Nutritional problems (underweight, overweight, obesity, coeliac disease)" |  |  |
